# Potato Fibers Have Positive Effects on Subjective Appetite Sensations in Healthy Men, but Not on Fecal Fat Excretion: A Randomized Controlled Single-Blind Crossover Trial

**DOI:** 10.3390/nu12113496

**Published:** 2020-11-13

**Authors:** Tanja Kongerslev Thorning, Christel Johanneson Bertolt, Mette S. Nielsen, Christian Ritz, Arne Astrup, Anne Raben

**Affiliations:** Department of Nutrition, Exercise and Sports, Faculty of Science, University of Copenhagen, 1958 Frederiksberg C, Denmark; tkthorning@gmail.com (T.K.T.); christel_johanneson@hotmail.com (C.J.B.); ritz@nexs.ku.dk (C.R.); ast@nexs.ku.dk (A.A.); ara@nexs.ku.dk (A.R.)

**Keywords:** dietary fibers, hunger, satiety, energy intake, gut metabolism, FiberBind, rhamnogalacturonan I

## Abstract

Dietary fibers can affect appetite and gut metabolism, but the effect of the novel potato fibers FiberBind and rhamnogalacturonan I (RG-I) is unknown. We, therefore, aimed to investigate the effect of daily intake of FiberBind and RG-I on appetite sensations and fecal fat excretion. In a single-blinded, randomized, three-way crossover trial, wheat buns with FiberBind, RG-I, or low fiber (control) were consumed by 18 healthy men during a 21-day period. Appetite sensation and blood samples during a 3 h meal test, fecal fat content, and ad libitum energy intake were assessed after each period. Compared to RG-I and control, FiberBind caused a higher composite satiety score (6% ± 2% and 5% ± 2%), lower prospective food consumption (5% ± 2% and 6% ± 2%), and lower desire to eat (7% ± 3% and 6% ± 3%) (all *p* < 0.05). FiberBind also caused higher satiety (6% ± 2%) and fullness (9% ± 3%) compared to RG-I (all *p* < 0.01). No effects on fecal fat excretion or energy intake were found. The RG-I fiber caused higher postprandial glucose concentration compared to FiberBind (*p* < 0.05) and higher insulin concentration at 180 min compared to control (*p* < 0.05). Compared to the control, RG-I and FiberBind lowered peak insulin concentration (both *p* < 0.05) and delayed time to peak for glucose (both *p* < 0.05). In conclusion, FiberBind intake could be beneficial for appetite regulation, but neither FiberBind nor RG-I affected fecal fat excretion or energy intake.

## 1. Introduction

The incidence of obesity is increasing worldwide. This is a major health concern since obesity is a risk factor for the development of various diseases, including type 2 diabetes, cancer, and cardiovascular diseases [1]. Dietary fibers may contribute to body weight management through the regulation of energy absorption and energy intake. Thus, some dietary fibers can affect energy absorption by increasing fecal fat excretion [2], and viscous fibers in particular have been shown to suppress appetite sensations and reduce subsequent energy intake [3,4]. However, decreased energy intake has also been shown after the intake of some insoluble fibers [5]. Dietary fibers may delay the gastric emptying rate, prolong the small intestine transit time, and thicken the unstirred water layer [6], thereby reducing the nutrient absorption rate [3,7,8], prolonging gut hormone release [9], and possibly increasing the satiety response.

Increased satiety could also be caused by an increased microbial fermentation of the fibers and short-chain fatty acids (SCFAs) production in the gut. SCFAs raise postprandial concentrations of the appetite-regulating hormones glucagon-like peptide 1 and peptide YY [10]. Further, dietary fibers reduce the energy density of foods/meals, and owing to the water binding capacity, cause stomach distension and afferent vagal signals of fullness [11]. Some viscous fibers are also potent in reducing risk markers associated with type 2 diabetes and cardiovascular diseases [12,13], i.e., blood pressure [14] and total and low-density lipoprotein (LDL)-cholesterol [15,16], and in prolonging the postprandial glucose and insulin response [17].

Potato pulp is a low-cost byproduct of the industrial production of potato flour (starch). It mainly consists of a plant cell wall material rich in dietary fibers, i.e., insoluble polysaccharides, cellulose, hemicellulose, resistant starch, and the soluble polysaccharides pectin [18]. The pectin fraction of potato pulp is especially rich in the viscous homogalacturonan (10–100 kDa) and rhamnogalacturonan I (RG-I) with long galactan side chains (>100 kDa) [18]. Viscous polysaccharides have been shown to impair the absorption of fat and reduce the reabsorption of bile acids [19]. The latter may affect lipid metabolism through increased de novo bile acid synthesis from hepatic cholesterol. Potato pulp per se was previously shown to have prebiotic properties and to reduce total and LDL-cholesterol in rats [20]. When fermented in vitro by microbiota from human feces, both homogalacturonan and RG-I were found to be bifidogenic [18].

To our knowledge, no studies have investigated whether these suggested effects of potato pulp and RG-I fiber also exist in humans. Therefore, the primary aim of the present study was to investigate the effect of three weeks of daily potato pulp fiber (FiberBind) or isolated RG-I intake vs. a low-fiber control on appetite and fecal fat excretion in healthy young men. We hypothesized that RG-I fiber increases satiety and fecal fat excretion compared to FiberBind and the control, and FiberBind increases satiety and fecal fat excretion compared to the control. Secondary aims were to investigate fecal energy excretion, fecal microbiota, body weight, blood lipids, insulin, glucose, a tight junction protein (zonulin), gastric emptying rate, colonic transit time, breath exhalation hydrogen, blood pressure, palatability, and tolerability.

## 2. Materials and Methods

### 2.1. Participants

A total of 25 normal to slightly overweight men were recruited from September 2016 to November 2016 through internet postings and recruitment notices at university campuses and other educational institutions in the Copenhagen area of Denmark. Inclusion criteria were: healthy, male gender, body mass index (BMI) 18.5–27.0 kg/m^2^, and 20–40 years of age. Reasons for exclusions were: potato intake with main meal >4 times per week, chronic diseases (known diabetes, cardiovascular disease, irritable bowels disease, colitis ulcerosa, Crohn’s disease, or other chronic diseases that could affect the results of the study), gluten allergy, use of daily prescription medicine (mild analgesics were allowed), use of lipid-lowering agents, use of food supplements of relevance to the study (such as pre- and probiotics), irregular intake of vitamin/mineral supplements, smoking, >10 h of strenuous physical activity per week, blood donation (<1 month before study commencement and during study period), or simultaneous participation in other clinical studies.

### 2.2. Design

**Figure 1:**
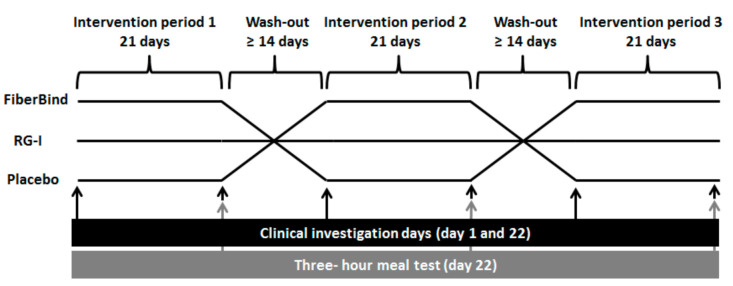
Study design. A randomized, three-way, crossover, placebo-controlled study with clinical investigations on Days 1 and 22 of each intervention period. RG-I, rhamnogalacturonan I.

The study was a single-blind randomized crossover trial with three intervention periods of 21 days each, separated by wash-out periods of at least 14 days (Figure 1). The participants were randomized to the sequence of interventions, using a computer-generated random sequence in the statistical program R. Participants and laboratory staff (but not researchers) were blinded until the study was completed and all laboratory analyses were finalized. Before the study commencement, participants completed a validated food frequency questionnaire used to assess their habitual dietary fiber intake [21]. During the intervention periods (Days 1–21), the participants consumed the allocated test product (i.e., test buns containing RG-I potato fibers (RG-I), potato pulp fibers (FiberBind), or buns without potato fiber (control)) along with their lunch and dinner meals. The participants kept a weighed food diary on Days 18–21 in the first intervention period, which was used as a menu plan for Days 18–21 in the following two intervention periods. This was done to standardize the participants’ habitual diets.

Before and after each intervention period (Days 1 and 22), after a 12 h overnight fast (0.5 L of water allowed) and a 24 h refrain from physical exercise and alcohol intake, the following were measured: body weight, blood pressure, and breath hydrogen (produced during colonic fermentation). On the same occasions, a fasting blood sample was drawn for analysis of total, LDL, and high-density lipoprotein (HDL)-cholesterol, triacylglycerol (TAG), insulin, glucose, and a tight junction marker (zonulin).

On examination Day 22, a three-hour meal test (2 megajoule (MJ) breakfast meal consisting of test buns with jam and 1500 mg of paracetamol) was carried out. In order to standardize meal intake prior to the meal test, participants consumed a standardized dinner meal together with one of the test buns before 8 p.m. the evening before the examination day. During the test meal, subjective appetite sensations were continuously registered for three hours (0, 15, 30, 16, 90, 120, 150, and 180 min). After 3 h, an ad libitum lunch meal was served and ad libitum energy intake was measured. Participants remained sedentary during the meal tests. In a random subgroup of nine participants, blood samples were drawn during the meal test subsequent to the appetite registrations (except 15 min), and these blood samples were analyzed for the concentrations of glucose, insulin, and paracetamol (a proxy for gastric emptying rate). In the last three days of the intervention periods (Days 19–21), the participants collected all of their feces, which were analyzed for the content of fat, energy, and microbiota. The defecation frequency and form of the feces during the three-day collection was self-assessed using the Bristol Stool Form Scale, as a proxy for colonic transit time [22,23].

Participants were instructed to maintain their habitual diet and level of physical activity during the study period and keep their intake of potatoes below twice a week in amounts below 200 g pr. time. Participants continuously registered their intake of potatoes.

The study was carried out at the Department of Nutrition, Exercise and Sports, Faculty of Sciences, University of Copenhagen, Frederiksberg, Denmark, from September 2016 to March 2017. The study was approved by the Ethical Committee of Copenhagen (H-16034271) and registered at clinicaltrials.gov as NCT02957318. All study participants gave written informed consent.

#### 2.2.1. The Intervention Fibers and the Control

FiberBind was a dietary fiber derived from potato pulp, consisting of 68% fiber. Of this fiber fraction, 5.5% was soluble, 76.7% was insoluble, and 17.8% was resistant starch (raw form). In addition, FiberBind contained 9.7% water, 0.3% fat, 7.2% protein, and 14% carbohydrate (starch). The energy content of FiberBind was 9.3 kJ/g (of this 5.44 kJ, ~58% was derived from fiber).

RG-I was a pectin fiber that was enzymatically extracted from FiberBind. RG-I contained 95% fiber and 5% water. Of the fibers, 100% was soluble. The energy content of RG-I was 7.6 kJ/g (of this 7.6 kJ, ~100% was derived from fiber).

The control was a low-fiber control matched for the energy and macronutrients provided from the FiberBind products.

All fibers were produced by KartoffelMelCentralen, Brande, Denmark.

#### 2.2.2. Test Buns

The test buns were energy- and macronutrient matched and designed using Dankost3000 dietary assessment software (Danish Catering Center). The potato fiber dose in the active intervention periods was 5 g/d (1 bun/d) during Week 1 and 10 g/d (2 buns/d) during Weeks 2 and 3 (Table 1). The increase in fiber after Week 1 was chosen to minimize potential gastrointestinal discomfort associated with a potential rapid increase in fiber intake.

#### 2.2.3. Standardized Dinner Meal

The evening before the visit on Day 22, participants consumed a standardized dinner meal along with one of the test buns before 8 p.m. The standardized dinner meal was equal for all participants and consisted of chicken curry with rice (3.5 MJ; 16.1 energy percentage (E%) protein, 33.6 E% fat, and 50.3 E% carbohydrate, 6 g of fibers).

#### 2.2.4. Breakfast Test Meal

The macronutrient distribution as well as the energy content and density of the standardized breakfast meals consumed in the three meal tests were similar (Table 2). The test meal consisted of the test buns (similar to those consumed in the just-completed 21-day intervention period) with jam and a glass of water and 1500 mg of paracetamol. The active test meals contained 10 g of fiber from FiberBind or RG-I. All test meals were matched for macronutrient composition, energy content, and energy density.

### 2.3. Outcomes

The coprimary outcomes were appetite and fecal fat excretion. The secondary outcomes were fecal energy excretion, fecal microbiota, body weight, blood lipids, insulin, glucose, a tight junction protein (zonulin), gastric emptying rate, colonic transit time, breath exhalation hydrogen, blood pressure, palatability, and tolerability.

#### 2.3.1. Appetite Sensation and Energy Intake

Visual analog scales (VAS) were used to assess subjective appetite sensations before the test meal (0 min) and at 15, 30, 60, 90, 120, 150, and 180 min after intake of the test meal. Furthermore, the palatability of the test meals was assessed at 15 min. VAS has previously been shown to be a valid and reproducible method for assessing subjective appetite sensations [24]. On each examination day (Day 22), participants received instructions on how to rate appetite sensations using VAS and were explained that the point registered should be the immediate sensations at each particular time point. The scales were based on a series of questions, each presented on individual sheets in a VAS booklet. The scale consisted of a 100 mm horizontal line with words anchored at each end, expressing the most positive and the most negative ratings of the appetite sensations: hunger, satiety, fullness, prospective food consumption, desire to eat, general wellbeing, and thirst, and of the palatability parameters: look, scent, taste, off-notes, and general appearance. The composite satiety score (CSS), a score indicating overall appetite, was calculated from the appetite sensations rated on VAS according to the equation: CSS (mm) = (satiety + fullness + (100 − prospective food consumption) + (100 − hunger)) / 4) [25].

Energy intake was assessed from an ad libitum lunch meal served 195 min after the test meal. The ad libitum method is validated to reflect spontaneous energy intake [26]. The meal consisted of spaghetti bolognese (8 MJ; 15.5 E% protein, 30.1 E% fat, and 54.5 E% carbohydrate). The participants were instructed to eat until they felt comfortably satiated. The time spent on consuming the ad libitum meal was registered.

#### 2.3.2. Feces Samples

All of the feces handed in by the participants were weighed. A fresh sample (<24 h) was drawn from one of the feces samples handed in during the three-day feces collection, and this sample was frozen at −80 °C. The fresh feces sample was analyzed for microbiota by 16S rDNA phylogenetic profiling. DNA was extracted from fecal samples using the 96-well NucleoSpin Soil DNA Isolation Kit (Macherey-Nagel). A minimum of one sample-well per plate was kept as a negative control during polymerase chain reaction (PCR) [27]. All plates also included a mock community to serve as an internal control for calibration of the bioinformatic pipeline. PCR was done with the forward primer S-D-Bact-0341-b-S-17 and reverse primer S-D-Bact-0785-a-A-21 with Illumina adapters attached [28]. The Illumina adapters are universal bacterial 16S rDNA primers, which target the V3-V4 region. The following PCR program was used: 98 °C for 30 s, 25× (98 °C for 10 s, 55 °C for 20 s, 72 °C for 20 s), and 72 °C for 5 min. Amplification was verified by running the products on an agarose gel. Indices were added in a subsequent PCR using the Nextera Index Kit V2 (Illumina) with the following PCR program: 98 °C for 30 s, 8× (98° C for 10 s, 55 °C for 20 s, 72 °C for 20 s), and 72 °C for 5 min. The attachment of barcodes was verified by running the products on an agarose gel. Products from the nested PCR were pooled and the resulting library cleaned with magnetic beads. The DNA concentration of pooled libraries was measured on a Qubit fluorometer using the Qubit High Sensitivity Assay Kit (Thermo Fisher Scientific). Sequencing was done on an Illumina MiSeq desktop sequencer using the MiSeq Reagent Kit V2 (Illumina) for 2× 300 bp paired-end sequencing. For each library, a mock community was included for calibration of the bioinformatics pipeline and to assess bias between sequencing runs.

Bioinformatics analysis: the 64-bit version of USEARCH [29] and mothur [30] were used in combination with several in-house programs for bioinformatics analysis of the sequence data. Following tag identification and trimming, all sequences from all samples were pooled. Paired-end reads were merged, truncating reads at a quality score of 4, requiring at least a 100 bp overlap and a merged read length between 300 and 600 bp in length. Sequences with ambiguous bases, without a perfect match to the primers, or a homopolymer length greater than 8 or with more than one expected errors were discarded. Sequences were strictly dereplicated, discarding clusters smaller than 5. Sequences were clustered at a 97% sequence similarity, using the most abundant strictly dereplicated reads as centroids and discarding suspected chimeras based on the internal comparison (USEARCH). Additional suspected chimeric OTUs were discarded based on comparison with the Ribosomal Database Project classifier training set v9 [31] using UCHIME [32]. The taxonomic assignment of OTUs was done using the method by Wang et al. [33] and the database from the Ribosomal Database Project.

All the collected feces, except for the fresh sample, were immediately frozen at −20 °C, freeze-dried, homogenized, and finally combined for each subject for each intervention period. The feces samples were acid hydrolyzed with 3M HCl at 80 °C for 1 h, after which fat content was measured by the method of Bligh and Dyer with modification [34,35]. A stepwise application of chloroform, methanol, and water was used to extract the lipids into the chloroform phase, and after evaporation, the fat content was determined gravimetrically in duplicates. Fecal energy content was measured by indirect calorimetry using a bomb calorimeter (ISO: 9831, Parr 6300 Oxygen Bomb Calorimeter, Parr Instrument Company, Moline, Il, USA).

#### 2.3.3. Colonic Transit Time

The Bristol Stool Form Scale, used as a proxy for colonic transit time, was self-assessed and registered by the participants for each of the collected feces samples during the three-day feces collection period. The mean feces form was calculated for each subject for each intervention period. The questionnaire has been validated to assess the stool form based on a 7-point scale [22].

#### 2.3.4. Gastrointestinal Symptoms

On Days 1, 7, 14, and 22 of each intervention period, the participants filled in a questionnaire on overall wellbeing and gastrointestinal symptoms, i.e., heartburn, acid reflux, bloating, stomach pain, nausea, stomach rumble, flatulence, constipation, diarrhea, and other symptoms. The symptoms were rated from 0 (non) to 4 (strong). A comparable questionnaire was also filled in after the ad libitum lunch meal on Day 22.

#### 2.3.5. Blood Samples

Fasting blood samples were drawn before and after the 21-day intervention periods. These samples were analyzed for blood lipids, i.e., total cholesterol, HDL-cholesterol, LDL-cholesterol, TAG, and glucose, insulin, and zonulin. Postprandial blood samples were drawn in a subgroup (*n* = 9) during the meal test at time points 15, 30, 60, 90, 120, 150, and 180. These samples were analyzed for insulin, glucose, and paracetamol.

Serum LDL-cholesterol and HDL-cholesterol were assessed with an enzymatic colorimetric procedure (ABX Pentra LDL Direct CP and ABX Pentra HDL Direct 100 CP, respectively). Serum total cholesterol and TAG concentrations were assessed by enzymatic procedures (CHOD-PAP and GPO-PAP, respectively). All analyses were carried out with a Pentra 400 Analyzer (Horiba ABX).

Serum insulin was measured with a solid-phase enzyme-labeled chemiluminescent immunometric assay using the Immulite 1000 Insulin kit (Siemens Medical Solution Diagnostics) and plasma glucose with ABX Pentra Glucose KH CP using ABX Pentra 400 (Horiba ABX, Cambridge Life Sciences, Cambridgeshire, UK).

The gastric emptying rate was assessed using paracetamol. Postprandial plasma samples were analyzed with the Paracetamol (Acetaminophen) Assay kit (Cambridge Life Science Ltd., Ely, Cambridgeshire, UK) using ABX Pentra 400 (Horiba ABX, Cambridge Life Sciences, Cambridgeshire, UK).

The tight junction marker zonulin was measured in serum with a Zonulin ELISA Kit (Immundiagnostik, Benshein, Germany) according to the manufacturer’s instructions.

#### 2.3.6. Anthropometry

Height without shoes was measured at screening to the nearest 0.5 cm using a wall-mounted Seca stadiometer (Seca, Hamburg, Germany). Fasting body weight was measured to the nearest 0.1 kg at screening, before and after the 21-day intervention periods on a Lindeltronic 800 scale, with participants wearing underwear and having emptied their bladder in advance.

#### 2.3.7. Blood Pressure

Fasting blood pressure was measured using an automatic sphygmomanometer before and after the 21-day intervention periods. It was measured 2 cm above the elbow bend on the arm after 10 min of rest in a reclined position. A cuff with the appropriate size was placed around the naked arm, three measurements were made, and the average of the last two measurements was used. There was no conversation during the blood pressure measurements, and the measurements were made on the same arm for each subject throughout the study.

#### 2.3.8. Breath Exhalation Hydrogen

Breath exhalation hydrogen was measured before and after the 21-day intervention periods in the fasted state, using a noninvasive Gastro+ Gastrolyzer (Bedfont Scientific Ltd., Kent, UK). The Gatrolyzer was calibrated with calibration gas, and the D-piece was changed on a monthly basis.

### 2.4. Statistical Analyses and Sample Size Calculation

The sample size calculation was based on previous data on the coprimary endpoints (appetite sensations and fecal fat excretion). Specifically, the detection of an effect size of 5% of mean values of the appetite ratings for satiety and hunger would require 18 participants with an 80% power at a 0.05 significance level [24]. Likewise, 18 participants would ensure a 99% power on a 0.05 significance level of detecting a 0.56 g difference in fecal fat excretion with a 0.31 standard deviation (SD) [2]. To take an expected 20% drop-out into account, 22 participants were planned to be included, and in the case of a drop-out rate higher than expected. Additional participants would be included to ensure sufficient statistical power.

Modified intention-to-treat analyses based on all available data from participants completing 2 or 3 periods were carried out. Linear mixed analysis of covariance (ANCOVA) models were fitted for the following outcome variables: fecal fat and fecal energy excretion, blood lipids, fasting insulin and fasting glucose, blood pressure, breath exhalation hydrogen, zonulin, body weight, energy intake, and colonic transit time. The models included subject-specific random effects. Diet and sequence were included as fixed effects. The baseline (Day 1) outcome values, when available, were included as fixed effects in the model. Additionally, the influence of body weight change was evaluated in the analyses of blood lipids, fasting insulin, and glucose and blood pressure.

Subjective appetite ratings and postprandial concentrations of insulin, glucose, and paracetamol, as well as gastrointestinal symptoms, were analyzed using linear mixed regression models with sequence, meal–time interaction, and 0 min time values as fixed effects and subject-specific random effects. Additionally, for the appetite sensations, the influence of palatability variables was also evaluated. Hence, approximate F tests were used to assess the overall effects of the diets, and stepwise model reduction was performed for nonsignificant variables in the model. However, the subject-specific random effect was kept in all models and so was the baseline outcome value. For the final models, *p*-values for the overall diet effects were reported, and when significant, pairwise comparisons between diets were subsequently performed using post hoc *t*-tests. In cases of significant meal-time interactions, the time points were separately explored.

Differences in alpha and beta diversity in microbiota were evaluated using pairwise *t*-tests. Identification of abundant differential bacteria by diet was performed using linear mixed regression models with subject-specific random effects, which were subsequently compared pairwise using Wilcoxon signed-rank test adjusted for subject id. The difference in *Bacteroidetes*/*Firmicutes* and *Bacteroides*/*Prevotella* ratio was evaluated using a Wilcoxon signed-rank test adjusted for subject id. Adonis (permanova) test was applied on the UniFrac distances for the data subsets and adjusted for subject id. The *p*-values were Bonferroni-adjusted to take into account the multiple comparisons.

Model assumptions were checked with a graphical assessment of residual plots and normal probability plots. If data were not normally distributed, they were transformed according to the best-fitted transformation. A value of *p* < 0.05 was declared significant.

All statistical analyses were performed using STATA v11.0 (StataCorp LP 2009, 4905 Lakeway Drive, College Station, TX USA), except for gut microbiota, which was analyzed using R v3.3.3.

## 3. Results

**Figure 2:**
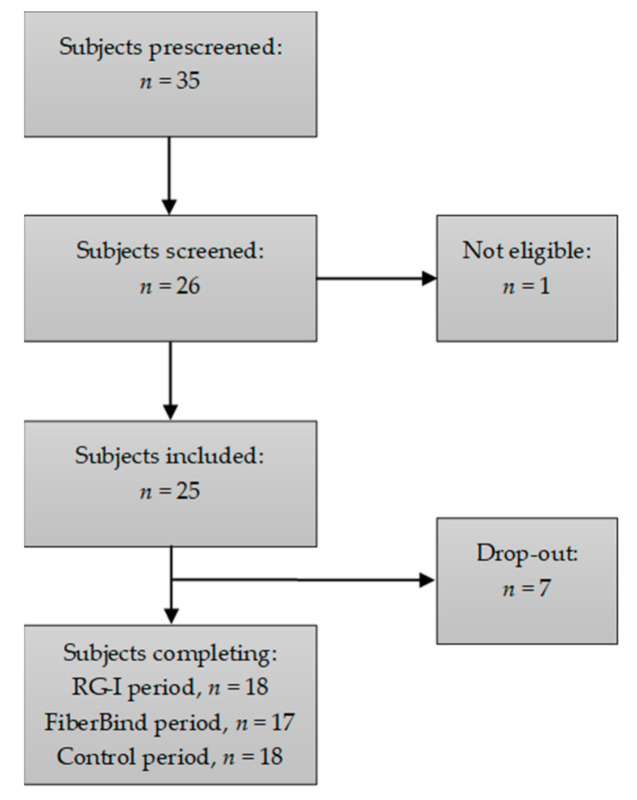
Subject flow chart.

A total of 17 out of the 25 included participants completed all three intervention periods, and one additional subject completed two intervention periods. Data from these 18 participants were included in the statistical analyses. The flow chart of the participants is shown in Figure 2. The baseline characteristics for the included participants are shown in Table 3. Reasons for drop-out were change of mind prior to start-up (*n* = 3), to time consuming/cumbersome (*n* = 2) or change of job or residential address (*n* = 2). During the study, the participants did not report any changes in physical activity or dietary intake, nor did they experience any adverse events related to the intake of the test buns.

### 3.1. Subjective Appetite Sensations during the Three-Hour Meal Test

There was a significant difference in ratings of satiety after intake of the three meal tests (*p* < 0.05). FiberBind caused higher satiety compared to RG-I (6% ± 2%, *p* < 0.01) but not the control (*p* = 0.07). Ratings of fullness were significantly different between the test meals (*p* < 0.01). Fullness was higher after FiberBind compared to RG-I (9% ± 3%, *p* < 0.01) but not the control (*p* = 0.08). Ratings of prospective food consumption, however, differed between the three test meals (*p* < 0.05), with a lower prospective food consumption after FiberBind compared with RG-I (5% ± 2%, *p* < 0.05) and the control (6% ± 2%, *p* < 0.05). There was a significant effect of the meals on ratings of desire to eat (*p* < 0.05). Desire to eat was lower after FiberBind compared to RG-I (7% ± 3%, *p* < 0.01) and the control (6% ± 3%, *p* < 0.05). Finally, the meals affected thirst differently (*p* < 0.05), with a higher thirst after FiberBind compared to RG-I (6% ± 3%, *p* < 0.05) and the control (6% ± 2%, *p* < 0.05).

**Figure 3:**
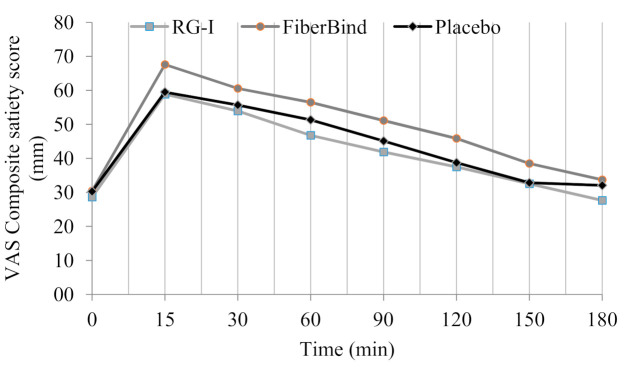
Mean ± standard error of mean (SEM) three-h repeated ratings of subjective appetite sensations presented as a composite satiety score (RG-I: *n* = 18, FiberBind: *n* = 17, control: *n* = 18). The composite satiety scorewas higher after FiberBind compared to RG-I (6% ± 2%, *p* < 0.05) and control (5% ± 2%, *p* < 0.05). Abbreviations: mm, millimeter; RG-I, rhamnogalacturonan I; VAS, visual analog scale.

For the CSS, there was a significant effect of the test meals (*p* < 0.05) (Figure 3). The overall CSS was higher after FiberBind compared to RG-I (6% ± 2%, *p* < 0.05) and the control (5% ± 2%, *p* < 0.05). No other differences in appetite ratings were observed.

### 3.2. Ad Libitum Energy Intake

No significant differences were found in energy intake at the *ad libitum* lunch meal (RG-I: 3847 kJ ± 224 kJ; FiberBind: 3966 kJ ± 333 kJ; Control: 3902 kJ ± 211 kJ). The palatability of the ad libitum lunch meal was rated above the median on all test days (non-significant (ns)).

### 3.3. Fecal Fat and Fecal Energy Excretion after the 21-Day Intervention Periods

Fecal fat excretion was 11.6 g/d ± 1.5 g/d with FiberBind, 10.6 g/d ± 1.1 g/d with RG-I, and 9.4 g/d ± 1.0 g/d with the control, with no significant effect of the intervention periods (*p* = 0.17). Likewise, there was no effect of the intervention periods on fecal energy excretion (*p* = 0.14). Fecal energy excretion was 1152 kJ/d ± 130 kJ/d with FiberBind, 1027 kJ/d ± 95 kJ/d with RG-I, and 962 kJ/d ± 93 kJ/d with the control.

### 3.4. Wellbeing during the Three-Hour Meal Test

There was no difference in wellbeing at 0 min. The ratings of wellbeing in the three hours after intake were not significantly different between the meals (RG-I: 64 mm ± 1 mm; FiberBind: 65 mm ± 1 mm; The control: 65 mm ± 1 mm).

### 3.5. Palatability of the Three Test Meals

Physical appearance, taste, and off-taste of the meals were rated significantly different between meals, with lower ratings for the FiberBind meal (Table 4). There was no significant difference in the assessment of the scent or overall palatability of the three meals.

### 3.6. Postprandial Glucose and Insulin Concentrations during the Three-Hour Meal Test

**Figure 4:**
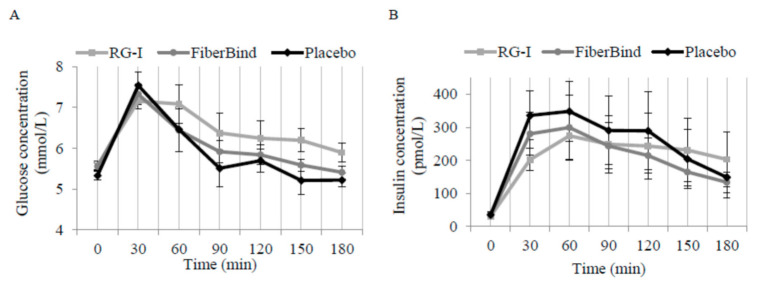
Mean ± SEM three-hour repeated measurements in a random subsample (*n* = 9) of (**A**) glucose concentrations and (**B**) insulin concentrations. Mean postprandial glucose was higher after RG-I compared to FiberBind (*p* < 0.01) but not the control. Insulin concentration was higher at 180 min after RG-I compared to the control (*p* < 0.05). Time to peak for glucose was delayed with the RG-I (*p* < 0.0001) and FiberBind (*p* < 0.05), compared to the control, and peak insulin concentration was also lower with RG-I (*p* < 0.01) and FiberBind (*p* < 0.05). Abbreviations: RG-I, rhamnogalacturonan I.

There was a significant effect of the meal tests on postprandial glucose concentrations (*p* < 0.05). The mean 0–180 min postprandial glucose was 0.33 ± 0.12 mmol/L higher after RG-I compared to FiberBind (*p* < 0.01) but not the control (0.25 mmol/L ± 0.14 mmol/L, *p* = 0.08) (Figure 4A). There was a significant effect of the meals on time to peak glucose concentration (*p* < 0.01). Compared to the control, the time to peak for glucose was 16 min ± 4 min delayed with RG-I (*p* < 0.0001) and 10 min ± 4 min delayed with FiberBind (*p* < 0.05).

An interaction between meal and time was detected for postprandial insulin concentration (*p* < 0.05). Thus, insulin concentration was 55% ± 17% higher at 180 min after RG-I compared to the control (*p* < 0.05) (Figure 4B). The peak insulin concentration was also different between the meals (*p* < 0.05). Compared to the control, peak insulin concentration was 103 pmol/L ± 37 pmol/L lower with RG-I (*p* < 0.01) and 82 pmol/L ± 37 pmol/L lower with FiberBind (*p* < 0.05).

### 3.7. Gastric Emptying Rate (Paracetamol) and Breath Hydrogen

Gastric emptying assessed by paracetamol concentrations did not differ between the meals (data not shown). The test buns affected breath exhalation hydrogen concentrations differently (*p* < 0.01) (Table 4), with a significantly higher breath exhalation hydrogen concentration after RG-I (change: 2 ppm ± 5 ppm, *p* < 0.001) and FiberBind (change: 8 ppm ± 3 ppm, *p* < 0.05) compared to the control (change −2 ppm ± 5 ppm).

### 3.8. Fecal Microbiota after the 21-Day Intervention Periods

**Figure 5:**
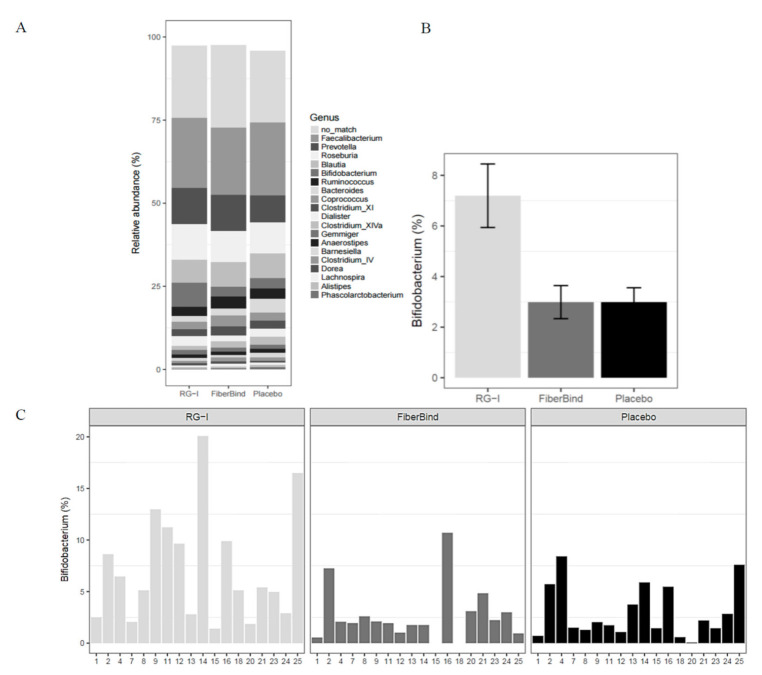
(**A**) Taxa summary plot at the genus level. Values illustrate mean values across the samples sorted according to the highest mean abundance. Only the 20 most abundant families and genera are shown. (**B**) Relative abundance (mean ± SEM) of the genera *Bifidobacterium* after three weeks of intervention with the three test buns (RG-I: *n* = 18, FiberBind: *n* = 16, control: *n* = 17). (**C**) Relative abundance of the genera *Bifidobacterium* per subject. Abbreviations: RG-I, rhamnogalacturonan I.

No differences in alpha diversity (diversity within the samples), beta diversity (diversity between the samples), or *Bacteroidetes*/*Firmicutes* and *Bacteroides*/*Prevotella* ratios were observed after the intervention periods (data not shown). The intervention periods did not induce large changes in the microbiota composition, as the main variation according to principal coordinates analysis (PCoA) plots was described by other factors than the test bun intake, such as subject id (plots not shown). However, when adjusting for the subject-specific variance using ADONIS (permanova) test, a small but significant effect was detected (*p* < 0.001), with a 2% difference between RG-I and the control (*p* < 0.01). When testing the 44 genera found to have a >1% abundance, there was a difference in the genera *Bifidobacterium* (*p* < 0.05), with a significantly higher relative abundance of *Bifidobacterium* with RG-I (7.2% ± 1.3%) compared to the control (3.0% ± 0.7%) and FiberBind (3.0% ± 0.6%) (*p* < 0.01 and *p* < 0.05, respectively) (Figure 5A,B). The effect of RG-I on *Bifidobacterium* was consistent among the participants (Figure 5C). No other differences in the abundance of other genus were found.

### 3.9. Other Secondary Outcomes after the 21-Day Intervention Periods

There was no significant difference in fasting concentrations of total cholesterol, HDL-cholesterol, LDL-cholesterol, TAG, insulin, or glucose, zonulin, colonic transit time, body weight, or blood pressure after the three-week intake of the test buns (Table 5).

Mean feces form, based on the seven-point scale (where “1” is very hard stool and “7” is very aqueous stools), was 3.9 ± 0.1 with RG-I, 3.8 ± 0.1 with FiberBind, and 3.8 ± 0.1 with the control (ns).

Gastrointestinal side effects were few and light during the intervention, with no significant differences during the three interventions (data not shown).

## 4. Discussion

To our knowledge, this is the first study in humans that investigates the effects of a daily intake of potato pulp fiber (FiberBind) and a novel soluble potato fiber (RG-I) on subjective appetite sensations and fecal fat excretion. FiberBind increased the postprandial feelings of satiety and reduced the feelings of hunger compared to RG-I fiber and the control. There were no significant differences in fecal fat or energy excretion between fibers and the control.

The finding of increased satiety after FiberBind was interesting but could not be explained by a difference in gastric emptying rate assessed by paracetamol. The rate of paracetamol absorption is known to depend predominantly on the rate of gastric emptying [36]. In our study, the postprandial plasma paracetamol, time to peak, as well as 0–180 min postprandial, were similar between the three meals. As appetite ratings were adjusted for differences in the palatability scores, these could also not explain the higher satiety and lower fullness scores with FiberBind. The satiating effect of FiberBind may, however, be due to the well-known bulking effect of the insoluble fiber [37], the fiber-type predominating in FiberBind. The higher satiety ratings after the FiberBind meal was not accompanied by a decrease in spontaneous energy intake at the following ad libitum meal. The time between the test meal and the ad libitum meal may have been too long to detect differences in energy intake, as the participants at 180 min after the meal were just as hungry as in the fasted state (0 min). Our study was not primarily designed for or powered to detect differences in spontaneous energy intake between the meals.

In our study, the time to peak glucose concentration was delayed after the fiber meals compared with the control meal, suggesting a slower release of glucose after the two potato fiber meals. This was also supported by a lower peak insulin concentration with the two potato fiber meals compared to the control meal. There was, however, no difference in peak glucose concentration between meals, so this suggests that less insulin was required to control the carbohydrate load consumed with the potato fiber meals. The lower peak insulin concentration and delay in time to peak glucose concentration were most pronounced with the RG-I meal. The overall 0–180 min postprandial glucose was higher for the RG-I meal than for the FiberBind and the control meals. Furthermore, insulin concentration was significantly higher at the last measurement (180 min) after the RG-I meal compared to the control, supporting a delayed glucose release, which persisted throughout the meal test. As there was no difference in the gastric emptying rate between the three test meals, this could not explain the differences in postprandial glucose and insulin kinetics after the meals. Some types of viscous dietary fiber, i.e., guar gum, psyllium, and β-glucan are known to form viscous gels in the gastrointestinal tract, thereby slowing down the absorption of glucose and causing reduced postprandial glycemia [38,39,40]. As observed for guar gum [41], it may be speculated that RG-I also produces a gel or mucilaginous layer around the starch granules in the gastrointestinal tract; however, this warrants further investigation.

The examination of breath exhalation hydrogen showed a significantly higher fasting breath hydrogen concentration after three weeks of intake of RG-I and FiberBind compared to the control. This finding confirms that the two potato fibers have reached the proximal colon undigested, causing increased bacterial fermentation. Endogenous hydrogen was recently suggested as a mediator between gut microbiota and host health, and specifically, microbiota-produced hydrogen may serve as a natural antioxidant protecting different tissues from free radicals [42]. In vitro, enzymatically solubilized potato pulp fiber was shown to have prebiotic properties when fermented by microbial communities derived from fecal samples from healthy humans [18]. In particular, solubilized potato fiber with a molecular mass >100 kDa, i.e., RG-I, was found to be three times more bifidogenic than fructooligosaccharides (FOS), a well-known prebiotic. Our in vivo study in humans confirmed a significantly ~4% higher relative abundance of *Bifidobacterium* in fecal microbiota after three weeks of intake of RG-I compared to the control. Different gut bacteria have more or less specificity with respect to energy substrates [43]. Based on the previous in vitro study, it was not expected that RG-I would affect the entire composition of the microbiota but rather influence specific bacteria genera. The higher abundance of *Bifidobacterium* after RG-I consumption is considered a health-promoting effect, as a low abundance of *Bifidobacterium* has been shown during old age, in childhood obesity, in women with a large weight gain during pregnancy, and in several diseases, i.e., irritable bowels syndrome, *Clostridium difficile-*associated diarrhea, cystic fibrosis, hepatitis B as well as type 1 and type 2 diabetes [44,45,46,47,48,49]. Bifidobacteria have been associated with the production of a number of health-promoting metabolites, including short-chain fatty acids, conjugated linoleic acid, and bacteriocins [50]. Furthermore, it was shown that a combination of prebiotics and bifidobacteria reduces the occurrence of carcinogen-induced cancerous cells in the gut through antimutagenic activity in rats [51]. It has also been shown to reduce colorectal proliferation and improve epithelial barrier function in cancer patients [52]. We did not observe differences in the tight junction protein zonulin between the intervention products, but it can be speculated that an improvement in gut barrier function is less likely in a population of healthy young men with presumably healthy guts.

No previous information was available on the potential gastrointestinal side effects of the potato fibers consumed in the current study. Therefore, caution was applied when determining the daily dose of potato fibers. Our data showed that a daily intake of 10 g of potato fibers does not cause gastrointestinal side effects and hence, is safe to consume for healthy adults.

The strengths of the current study were its strong crossover design and that it strived to imitate real-life settings. Limitations of the present study include that it was not possible to blind the test buns to the researchers. However, the participants and laboratory technicians conducting the blood analyses were not aware of the test bun allocation sequence. The fiber doses in our study may also have been too low to induce health effects aside from the change in gut microbiota, as previous studies have suggested dose-dependent effects of other prebiotics on the gut microbiota shift and change in metabolic markers [50]. Furthermore, the intervention durations in our study may have been too short to induce the full potential of RG-I on gut microbiota and its metabolites, and the inclusion of healthy participants may explain the lack of effects on other health markers measured. Hence, we cannot predict the effects in the longer term, with higher fiber doses or in participants with increased disease risk markers. Finally, we only enrolled healthy male participants. Thus, the results may not be generalizable to other populations.

## 5. Conclusions

FiberBind had a beneficial effect on appetite regulation, delayed the glucose peak, and lowered the insulin peak. RG-I also delayed glucose peak and prolonged the glucose response, which had not returned to baseline after 3 hours. This was supported by a higher insulin concentration after 3 hours compared to the control. The potato fibers did not significantly affect fecal fat excretion or energy intake compared to the control, but FiberBind and RG-I increased breath exhalation hydrogen concentrations and RG-I acted prebiotic as seen by the increased relative abundance of *Bifidobacterium* in the gut. This can be considered a beneficial health effect.

## Figures and Tables

**Table 1:** Composition of the three test buns.

Test Buns	Carbohydrate (E%)	Protein (E%)	Fat (E%)	Fiber (g)	Energy Per Bun (KJ)
RG-I	85.4	10.7	3.9	7.2	874
FiberBind	84.7	11.4	3.9	7.1	874
Control	85.4	10.7	3.9	2.2	873.7

RG-I: buns containing RG-I potato fibers (recipe per bun: 5.3 g of RG-I product, 56 g of wheat flour, 36 g of water, 1.1 g of salt, 1.5 g of yeast). FiberBind: buns containing potato pulp fibers (recipe per bun: 7.4 g of FiberBind product, 54.14 g of wheat flour, 65 g of water, 1.1 g of salt, 4.0 g of yeast). Control: buns without potato fiber (recipe per bun: 56 g of wheat flour, 2.76 g of potato flour, 39 g of water, 1.1 g of salt, 1.5 g of yeast). Abbreviations: E%, energy percentage; RG-I, rhamnogalacturonan I; kJ, kilojoule.

**Table 2:** Composition of the breakfast test meals.

Breakfast Test Meals	Carbohydrate		Protein		Fat		Fiber	Energy
	(E%)	(g)	(E%)	(g)	(E%)	(g)	(g)	(kJ)
RG-I meal	80.5	88.0	9.4	11.0	10.1	5.5	14.4	2000
FiberBind meal	80.0	87.4	10.0	11.7	10.1	5.4	14.3	2000
Control meal	80.5	92.7	9.4	11.0	10.1	5.5	4.3	2000

RG-I meal: A meal with 2 test buns containing RG-I potato fibers. FiberBind meal: A meal with 2 test buns containing potato pulp fibers. Control meal: A meal with 2 control test buns without potato fiber. All meals were served with 4.4 g of butter, 20 g of jam sweetened with stevia, and 250 g of water. Abbreviations: E%, energy percentage; g, grams; kJ, kilojoule; RG-I, rhamnogalacturonan I.

**Table 3:** Subject characteristics at inclusion.

Age (y)	26.0 ± 4.7
Body weight (kg)	76.6 ± 7.1
Height (m)	1.81 ± 0.1
BMI (kg/m²)	23.2 ± 1.6
Habitual fiber intake (g/d)	29.0 ± 14.6

Data reported as means ± standard deviation (SD) (*n* = 25). Abbreviations: y, years; d, day.

**Table 4:** Palatability ratings of the three test meals.

Palatability	RG-I ^1^	FiberBind ^2^	Control ^1^	*p_d_*_iet_
Physical appearance of test meal (mm)	53.4 ± 5.2 ^ab^	50.6 ± 4.0 ^a^	61.7 ± 3.6 ^a^	0.03
Taste of test meal (mm)	62.3 ± 3.6 ^a^	51.6 ± 4.1 ^b^	64.2 ± 3.6 ^a^	0.03
Scent of test meal (mm)	60.2 ± 4.3	61.2 ± 3.6	66.2 ± 2.7	0.18
Off-taste of test meal (mm)	6.3 ± 1.5 ^a^	17.6 ± 5.5 ^b^	10.1 ± 2.1 ^a^	0.03
Overall palatability of test meal (mm)	60.9 ± 4.9	55.9 ± 3.5	67.0 ±3.6	0.06

RG-I: meal with buns containing RG-I potato fibers. FiberBind: meal with buns containing potato pulp fibers. Control: meal with buns without potato fiber. ^1^ *n* = 18, ^2^ *n* = 17. Data are shown as means ± standard error of mean (SEM), *p*_diet_ reports the overall effect by using an analysis of covariance (ANCOVA), with subject ID as a random variable. Numbers with different letters (i.e., a, b) are significantly different (*p* < 0.05). Abbreviations: mm, millimeter; RG-I, rhamnogalacturonan I.

**Table 5:** Fasting parameters on Days 1 and 22 of the three intervention periods.

Fasting Parameters	RG-I	FiberBind	Control	*p*_diet_
Body weight (kg)				
Day 1	76.2 ± 1.7	75.8 ± 1.7	77.1 ± 1.7	
Day 22	76.2 ± 1.7	76.3 ± 1.8	77.5 ± 1.8	0.19
Systolic blood pressure (mmHg)				
Day 1	125.0 ± 1.6	127.1 ± 1.9	123.9 ± 1.9	
Day 22	122.8 ± 1.3	123.6 ± 1.8	123.4 ± 2.0	0.75
Diastolic blood pressure (mmHg)				
Day 1	70.6 ± 1.5	70.8 ± 2.2	70.2 ± 1.9	
Day 22	68.4 ± 2.1	69.2 ± 2.0	67.8 ± 1.9	0.86
Total cholesterol (mmol/L)				
Day 1	4.18 ± 0.24	4.36 ± 0.25	4.37 ± 0.27	
Day 22	4.12 ± 0.25	4.25 ± 0.28	4.02 ± 0.24	0.09
LDL-cholesterol (mmol/L)				
Day 1	2.53 ± 0.21	2.56 ± 0.21	2.65 ± 0.23	
Day 22	2.48 ± 0.23	2.61 ± 0.25	2.44 ± 0.20	0.14
HDL-cholesterol (mmol/L)				
Day 1	1.35 ± 0.06	1.48 ± 0.08	1.42 ± 0.09	
Day 22	1.33 ± 0.08	1.32 ± 0.07	1.29 ± 0.07	0.16
TAG (mmol/L)				
Day 1	1.00 ± 0.15	1.06 ± 0.20	0.92 ± 0.13	
Day 22	0.94 ± 0.12	0.92 ± 0.13	0.92 ± 0.12	0.54 ^1^
Insulin (pmol/L)				
Day 1	37.6 ± 6.3	44.4 ± 6.2	35.3 ± 5.9	
Day 22	27.6 ± 4.0	31.2 ± 6.2	31.7 ± 4.8	0.39 ^1^
Glucose (mmol/L)				
Day 1	5.60 ± 0.08	5.61 ± 0.07	5.45 ± 0.06	
Day 22	5.50 ± 0.08	5.50 ± 0.07	5.43 ± 0.08	0.85
Breath Exhalation Hydrogen (ppm)				
Day 1	20.4 ± 6.2	8.9 ± 2.2	16.6 ± 3.6	
Day 22	22.7 ± 3.0 ^a^	17.0 ± 2.7 ^a^	14.6 ± 3.4 ^b^	0.001 ^1^
Zonulin (ng/mL)				
Day 1	54.8 ± 3.3	55.9 ± 3.3	53.9 ± 2.8	
Day 22	51.6 ± 2.8	52.3 ± 3.1	52. 1 ± 3.1	0.59

RG-I: buns containing RG-I potato fibers. FiberBind: buns containing potato pulp fibers. Control: control buns without potato fiber. Raw means ± SEMs are shown. *p*_diet_ is based on linear mixed analysis of covariance (ANCOVA) models. The models included subject-specific random effects and diet and sequence as fixed effects. The baseline (Day 1) values were included as fixed effects in the models. ^1^ *p*-values are based on transformed variables (both *F*-test and the pairwise comparisons). Numbers with different letters (i.e., a, b) are significantly different (*p* < 0.05). Abbreviations: HDL, high-density lipoprotein; LDL, low-density lipoprotein; RG-I, rhamnogalacturonan I; TAG, triacylglycerol.
